# Mesenchymal Stromal Cells Express GARP/LRRC32 on Their Surface: Effects on Their Biology and Immunomodulatory Capacity

**DOI:** 10.1002/stem.1821

**Published:** 2014-12-18

**Authors:** Ana Belén Carrillo-Galvez, Marién Cobo, Sara Cuevas-Ocaña, Alejandra Gutiérrez-Guerrero, Almudena Sánchez-Gilabert, Pierpaolo Bongarzone, Angélica García-Pérez, Pilar Muñoz, Karim Benabdellah, Miguel G Toscano, Francisco Martín, Per Anderson

**Affiliations:** GENYO, Centre for Genomics and Oncological Research, Pfizer/University of Granada/Andalucian Regional GovernmentPTS Granada, Granada, Spain

**Keywords:** Glycoprotein A repetitions predominant, Leucine-rich repeat containing 32, Mesenchymal stem cells, Membrane bound TGF-β1, Proliferation, Immunomodulation

## Abstract

Mesenchymal stromal cells (MSCs) represent a promising tool for therapy in regenerative medicine, transplantation, and autoimmune disease due to their trophic and immunomodulatory activities. However, we are still far from understanding the mechanisms of action of MSCs in these processes. Transforming growth factor (TGF)-β1 is a pleiotropic cytokine involved in MSC migration, differentiation, and immunomodulation. Recently, glycoprotein A repetitions predominant (GARP) was shown to bind latency-associated peptide (LAP)/TGF-β1 to the cell surface of activated Foxp3^+^ regulatory T cells (Tregs) and megakaryocytes/platelets. In this manuscript, we show that human and mouse MSCs express GARP which presents LAP/TGF-β1 on their cell surface. Silencing GARP expression in MSCs increased their secretion and activation of TGF-β1 and reduced their proliferative capacity in a TGF-β1-independent manner. Importantly, we showed that GARP expression on MSCs contributed to their ability to inhibit T-cell responses in vitro. In summary, we have found that GARP is an essential molecule for MSC biology, regulating their immunomodulatory and proliferative activities. We envision GARP as a new target for improving the therapeutic efficacy of MSCs and also as a novel MSC marker. Stem Cells *2015;33:183–195*

## Introduction

Mesenchymal stromal cells (MSCs) are nonhematopoietic, multipotent cells with self-renewal capacity present in virtually all tissues. Their secretion of growth factors and potent immunomodulatory properties make them a promising tool for therapy in inflammatory/autoimmune diseases. Although MSC therapy is deemed safe, several phase III clinical trials using MSCs for the treatment of inflammatory/autoimmune diseases have failed to show any significant clinical benefit 1,2. It has become clear that we need a better characterization of the MSC preparations and a better understanding of MSC biology, including the mechanisms behind their immunomodulatory capacity.

The transforming growth factors-β (TGF-β1, 2, and 3) are pleiotropic molecules involved in multiple biological processes including development, control of stem cell behavior, carcinogenesis, tissue homeostasis and regeneration, and immune responses 3–5. TGF-β1 is produced by MSCs and has been demonstrated to promote fibroblast proliferation and to participate in MSC-mediated immunomodulation 6–8. TGF-β1 also directly influences MSCs, affecting both their migration and differentiation 9,10 highlighting the importance of TGF-β1 for the therapeutic activity of MSCs. The TGF-βs are produced as precursor peptides consisting of the mature TGF-β domain and the latency-associated peptide (LAP) domain. The precursor peptide is cleaved by the endoprotease furin, thus separating the two domains and LAP then associates noncovalently with the mature TGF-β peptide forming the small latency complex (SLC). LAP maintains TGF-β biologically inactive and its removal enables TGF-β to bind to its receptors and induce signaling 11. Inside the cell, the SLC normally associates with latent TGF-β binding proteins (LTBPs) forming the large latency complex, which is then secreted and anchored to the extracellular matrix (ECM) 12,13 where TGF-β can be activated by reactive oxygen species, low pH, integrins, proteases, and thrombospondin-1 14. Recently, glycoprotein A repetitions predominant (GARP), also called leucine-rich repeat containing 32 (LRRC32) 15, was shown to bind LAP/TGF-β1 and present it on the cell membrane of platelets and activated CD4^+^Foxp3^+^ regulatory T cells (Tregs) 16,17. Several cell types express membrane bound TGF-β1 (mbTGF-β1) including immature dendritic cells and myeloid-derived suppressor cells 18–20 but GARP expression has so far only been described on the surface of activated Tregs and megakaryocytes/platelets 17,21. GARP regulates the bioavailability of TGF-β1 and its role has primarily been studied in the context of T-cell differentiation and function where GARP-bound LAP/TGF-β1 on Tregs is involved in both infectious tolerance and the induction of T-helper (Th)−17 cells 22,23. Recently, expression of mbTGF-β1 was found on human umbilical cord blood-derived MSCs 24 and on microvesicles prepared from murine bone marrow-derived MSCs (BM-MSCs) 25. However, how TGF-β1 is attached to the cell surface of MSCs and the role of mbTGF-β1 in MSC biology and therapeutic efficacy are unknown. In this report, we show that GARP is expressed on the surface of mouse and human MSCs colocalizing with mbTGF-β1. Silencing of GARP in MSCs reduced the surface expression of LAP/TGF-β1 while increasing its secretion and activation. Interestingly, MSCs lacking GARP exhibited an impaired proliferative capacity and were less efficient in suppressing the proliferation of T cells in vitro. In summary, this study highlights the importance of GARP for MSC biology and function, adding a new molecule that can be modulated when aiming to improve the therapeutic efficacy of MSCs for the treatment of autoimmune/inflammatory diseases.

## Materials and Methods

### Animals

Male BALB/c (6–10 weeks; Charles River, Barcelona, Spain, http://www.criver.com) were used to initiate cultures of mASCs and mBM-MSCs or for the generation of splenocyte cell suspensions. All experiments were performed according to the Institutional Guidelines for the Care and Use of Laboratory Animals in Research and with the approval of the local ethics committee at the H. U. Virgen Macarena in Seville, Spain.

### Isolation and Culturing of MSCs

Abdominal (epididymal) and subcutaneous (inguinal) fat from BALB/c mice (Charles River) were processed as previously described 26. Cells were resuspended in complete MesenCult medium (Stem Cell, Grenoble, France, http://www.stemcell.com) and seeded at a density of 20,000–30,000 cells per square centimeter and cultured at 5% O_2_/5% CO_2_ at 37°C. Subsequent passages were plated at 10,000 cells per square centimeter. BM-MSCs were obtained by flushing femurs and tibiae of male BALB/c mice and adding 1.5 × 10^6^ cells per square centimeter in cell culture flasks. Human ASCs were obtained from Biobanco del Sistema Sanitario Público de Andalucía (Granada, Spain) and cultured in advanced Dulbecco's modified Eagle's medium (DMEM) supplemented with 10% fetal calf serum (FCS) (Invitrogen, Carlsbad, CA, http://www.lifetechnologies.com), Glutamax (GIBCO, Grand Island, NY, http://www.lifetechnologies.com), and 100 U/ml penicillin/streptomycin (GIBCO). The phenotypic characterization and differentiation of mASCs into adipocytes, osteocytes, and chondrocytes were performed as previously described 27 (Supporting Information Fig. S1). All experiments using human samples were performed according to the Institutional Guidelines and approved by the ethics committee of the H.U. Virgen de Macarena.

### Lentiviral Vector Production and ASC Transduction

MISSION shRNA plasmid DNAs specific for mouse GARP (LRRC32) (RefSeq: SHCLND-NM_001113379; two different mouse GARP/LRRC32-specific shRNA plasmids were used, referred to in the manuscript as LV#3 and LV#6), for human GARP (RefSeq: SHCLND-NM_005512; two different human GARP/LRRC32-specific shRNA plasmids were used, referred to in the manuscript as LV#18 and LV#19) and a MISSION pLKO.1-puro nonmammalian shRNA control plasmid (all from Sigma Aldrich, St Louis, MO, http://www.sigmaaldrich.com) were amplified using One Shot Stbl3 *Escherichia coli* (Invitrogen) grown at 30°C. Lentiviral vectors (LVs) were produced by cotransfecting 293T cells with: (a) vector shRNA plasmid, (b) packaging plasmid pCMVΔR8.91, and (c) envelope plasmid pMD.G, using LipoD In Vitro DNA Transfection Reagent (Ver. II; SignaGen Laboratories, Rockville, MD, http://www.signagen.com) and concentrated as previously described 28. For transduction of ASCs, 0.7 × 10^6^ ASCs (passages 2–4) were mixed with the concentrated virus, left at room temperature for 10 minutes, and subsequently seeded in six-well plates and maintained at 5% O_2_; 5% CO_2_ at 37°C for 5 hours. Cells were then washed, seeded in T75 flasks, and incubated at 5% O_2_; 5% CO_2_ at 37°C. GARP expression was assayed by flow cytometry and RT-qPCR on days 3 and 5 after transduction, respectively. Vector copy number per transduced ASC was determined by qPCR, using the QuantiTect SYBRGreen PCR kit (Qiagen, Hilden, Germany, http://www.qiagen.com), performed on an MX3005Pro sequence detection system (Stratagene, La Jolla, CA, http://www.stratagene.com) as previously described 29. For the different LV-transduced cells, the following primers were used: puromycin FW: 5′-TGCAAGAACTCTTCCTCACG-3′, puromycin RV: 5′-AGGCCTTCCATCTGTTGCT-3′. Tenfold increasing amounts of plasmid DNA (10^2^ up to 1 × 10^7^ copies) were used to determine the standard curve in each experiment.

### Detection of Surface and Intracellular GARP and LAP/TGF-β1 Expression

For LAP/TGF-β1 staining, mASCs were plated at 5,000 cells per square centimeter and after 24–48 hours cells were harvested using phosphate buffered saline (PBS) with 2 mM EDTA. Cells were incubated with 7AAD (Sigma-Aldrich) and 2.4G2 (for mASCs; eBioscience, San Diego, CA, http://www.eBioscience.com) followed by anti-mouse LAP/TGF-β1 (TW7-16B4) or anti-human LAP/TGF-β1 (TW4-6H10) (Biolegend, San Diego, CA, http://www.biolegend.com) followed by goat anti-mouse IgG-APC (Jackson Immunoresearch, West Grove, PA, http://www.jacksonimmuno.com) or a donkey anti-mouse IgG-Alexa488 (Molecular Probes, Carlsbad, CA, http://www.lifetechnologies.com), respectively. For GARP expression, ASCs were harvested using TrypLE (Gibco) and stained for murine GARP (Garp-PE; YGIC86), with or without Sca-1, or human GARP (GARP-eFluor660; G14D9) all from eBioscience. For GARP staining of human platelets, blood from healthy volunteers was collected in EDTA tubes and centrifuged at 400*g* for 7 minutes to obtain the platelet-containing supernatant. Platelets were then precipitated at 800*g* for 7 minutes and washed with PBS, centrifuged again at 400*g* to discard cellular contaminants and counted. 10^6^ human platelets were then stained for human GARP (GARP-eFluor660; G14D9) and CD41a-PE (HIP8; eBioscience). For intracellular staining of GARP, ASCs were fixed, permeabilized, and stained using the BD Cytofix/Cytoperm kit according to the manufacturer's instructions (BD Biosciences, San Diego, CA, http://www.bdbiosciences.com). Cells were acquired on a FACS Canto II flow cytometer and analyzed using the FACS Diva software (BD Biosciences). Corresponding isotype controls were used for determining background staining.

### mRNA Analysis by RT-qPCR

Total RNA was obtained using the Trizol reagent (Invitrogen) according to the manufacturer's instructions. RNA samples were reverse-transcribed using the Superscript first-strand system (Invitrogen) and qPCRs were performed using the QuantiTect SYBRGreen PCR kit (Qiagen) on a Stratagene MX3005P system (Agilent Technologies, Santa Clara, CA, http://www.agilent.com). Mouse-specific Primers: GARP FW: 5′-ACCAGATCCTGCTACTCCTG-3′, GARP RV: 5′-ACGAAGCGCTGTATAGAAGC-3′; TGF-β1 FW: 5′-TGCGCTTGCAGAGATTAAAA-3′, TGF-β1 RV: 5′-AGCCCTGTATTCCGTCTCCT-3′; IL-11 FW: 5′-TCCTTCCCTAAAGACTCTGG-3′, IL-11 RV: 5′-TTCAGTCCCGAGTCACAGTC-3′; cnn-1 FW: 5′-ACAAGAGCGGAGATTTGAGC-3′, cnn-1 RV: 5′-TGAGTGTGTCGCAGTGTTCC-3′; HES1 FW: 5′-CGGCATTCCAAGCTAGAGAAGG-3′, HES1 RV: 5′-GGTAGGTCATGGCGTTGATCTG-3′; β-actin FW: 5′-AATCGTGCGTGACATCAAAG-3′, β-actin RV: 5′-ATGCCACAGGATTCCATACC-3′. Human-specific primers: GARP FW: 5′-ACAACACCAAGACAAAGTGC-3′, GARP RV: 5′-ACGAAGTGCTGTGTAGAAGC-3′; IL-11 FW: 5′-GACCTACTGTCCTACCTGCG-3′, IL-11 RV: 5′-AGTCTTCAGCAGCAGCAGTC-3′; GAPDH FW: 5′-ATGGGGAAGGTGAAGGTCG-3′, GAPDH RV: 5′-GGGGTCATTGATGGCAACAATA-3′.

### Western Blot

Cells were lysed in RIPA buffer containing protease inhibitors (both from Sigma Aldrich) at 1 × 10^6^ cells/100 µl. The lysates (20 µg/sample) were resolved by sodium dodecyl sulfate-polyacrylamide gel electrophoresis (10% polyacrylamide under reducing conditions) and electrotransferred to nitrocellulose membranes (Bio-Rad, Hercules, CA, http://www.bio-rad.com). Membranes were blocked with 5% nonfat milk and probed with anti-LRRC32 (Plato-1; ENZO Life Sciences, Ann Arbor, MI, http://www.enzolifesciences.com) at 1:500 dilution at 4°C overnight, followed by 1 hour of incubation with goat anti-mouse IgG-IRDye680 (1:20,000 dilution; LI-COR Biosciences, Lincoln, NE, http://www.licor.com). Protein was detected using an Odyssey Image scanner system (LI-COR Biosciences).

### Confocal Microscopy

For LAP/GARP costaining, mASCs were harvested with EDTA as described above and stained with GARP-PE (YGIC86; eBioscience) and purified anti-mouse LAP/TGF-β1 (TW7–16B4) followed by a donkey anti-mouse IgG-Alexa488 (Molecular Probes). For phospho-Smad2/3 staining, NT, CTRL-LV, LV#3, and LV#6 mASCs were plated in Lab-Tek II cc^2^ eight-well chamber slides (Nalgene, Thermo Fisher Scientific, Waltham, MA, http://www.thermofisher.com) at 5,000 cells/well and cultured in MesenCult supplemented with 0.2% FCS for 2 days. Cells were stained for phospho-Smad2/3 (rabbit anti-mouse/human phospho-Smad2 [Ser465/467]/Smad3 [Ser423/425]; Cell Signaling Technologies, Beverly, MA http://www.cellsignal.com) followed by a goat anti-rabbit Alexa555 Ab (Molecular Probes) according to the manufacturer's protocol. Slides were mounted using Slowfade Gold antifade reagent containing DAPI (Molecular Probes). Images were acquired on a Zeiss LSM 710 confocal microscope (Carl Zeiss, Jena, Germany, http://www.zeiss.com). The extent of colocalization (overlapping coefficient according to Mander) was calculated using the ZEN 2010 software (Carl Zeiss).

### Analysis of mASC Proliferation

Proliferation of ASCs was measured using the xCelligence real-time cell analyzer system from Roche (Roche Applied Science, Penzberg, Germany, http://www.roche.com). In brief, 800 cells/well of NT, LV-CTRL, LV#3, and LV#6 mASCs or NT, LV-CTRL, LV#18, and LV#19 hASCs were added to 16-well E-plates as described previously 30. The E-plates were then placed on the device station in the incubator (normoxia; 5% CO_2_ at 37°C) for continuous recording of impedance, as reflected by cell index. In some experiments, a TGF-β1/2/3-neutralizing antibody (1D11; R&D Systems, Minneapolis, MN, http://www.rndsystems.com) or SB431542 (Sigma) was added on days 1 and 3 to the wells at 2.5 µg/ml and 10 µM, respectively. Cell proliferation was also assayed using the CellTiter-Blue reagent, according to the manufacturer's instructions (Promega, Madison, WI, http://www.promega.com). In brief, NT, LV-CTRL, LV#3, and LV#6 mASCs were seeded in 96-well plates (800 cells/well) and cultured at 5% O_2_/5% CO_2_ at 37°C. On days 1, 3, and 7, CellTiter-Blue was added to the cells during the last 4 hours of culture and fluorescence was measured at 560 nm on a Glomax multidetection system (Promega).

### Assessment of the Immunomodulatory Activity of mASCs In Vitro

Cocultures of carboxyfluorescein succinimidyl ester (CFSE)-labeled splenocytes and mitomycin C-treated mASCs (NT, LV-CTRL, LV#3, and LV#6) were set up as previously described 27. Cell division was analyzed using the FlowJo software (Tree Star, Inc., Ashland, OR, http://www.flowjo.com). Culture supernatants were assayed for NO_2_ content using the Griess assay as previously described 27. In some mASC:splenocyte cocultures, Compound E (Enzo Life Sciences) were added at 50, 100, and 500 nM. For analyzing the expression of HES1 in responder T cells, ASC:splenocyte cocultures were set up as described above. After 1 day of stimulation, the splenocytes were collected and used for RT-qPCR for HES1. Splenic natural killer (NK) cells were purified using the NK cell isolation kit II (Miltenyi Biotech, Bergisch Gladbach, Germany, http://www.miltenyibiotech.com) according to the manufacturer's instructions. NK cells (60,000 cells) were added to mitomycin C-treated NT, LV-CTRL, LV#3, or LV#6 mASCs (60,000 cells) in 24-well plates and cultured for 5 days in the presence or absence of 100 ng/ml recombinant murine IL-15 (Peprotech, Rocky Hill, NJ, http://www.peprotech.com). The nonadherent cells in the cocultures were collected and stained for CD49b and NKG2D (both from eBioscience) and analyzed on a FACS Canto flow cytometer (BD Biosciences). RPMI with 10% heat-inactivated serum was used for both coculture assays.

### Measurement of TGF-β1 Production by ASCs

NT, LV-CTRL, LV#3, and LV#6 mASCs or NT, LV-CTRL, LV#18, and LV#19 hASCs (20,000 cells per square centimeter) were cultured for 48 hours and the TGF-β1 levels in supernatants, w/ or w/o acid activation, were analyzed using a TGF-β1 Ready-SET-go ELISA kit (eBioscience) according to the manufacturer's description. The TGF-β1 levels in complete MesenCult and advanced DMEM media were subtracted from the data obtained from the ASC cultures.

### Statistical Analysis

Statistical analysis was performed using the GraphPad Prism software (GraphPad Software, Inc., La Jolla, CA, http://www.graphpad.com). All data are represented as mean (SEM) of three independent experiments unless otherwise stated in the figure legend. Pairwise and multiple comparisons of the data have been performed using the nonparametric Mann-Whitney *U* test and the Kruskal-Wallis test followed by the Dunn's post-test, respectively. *p* values <.05 were considered statistically significant.

## Results

### MSCs Express GARP and LAP/TGF-β1 on Their Surface

**Figure 1:**
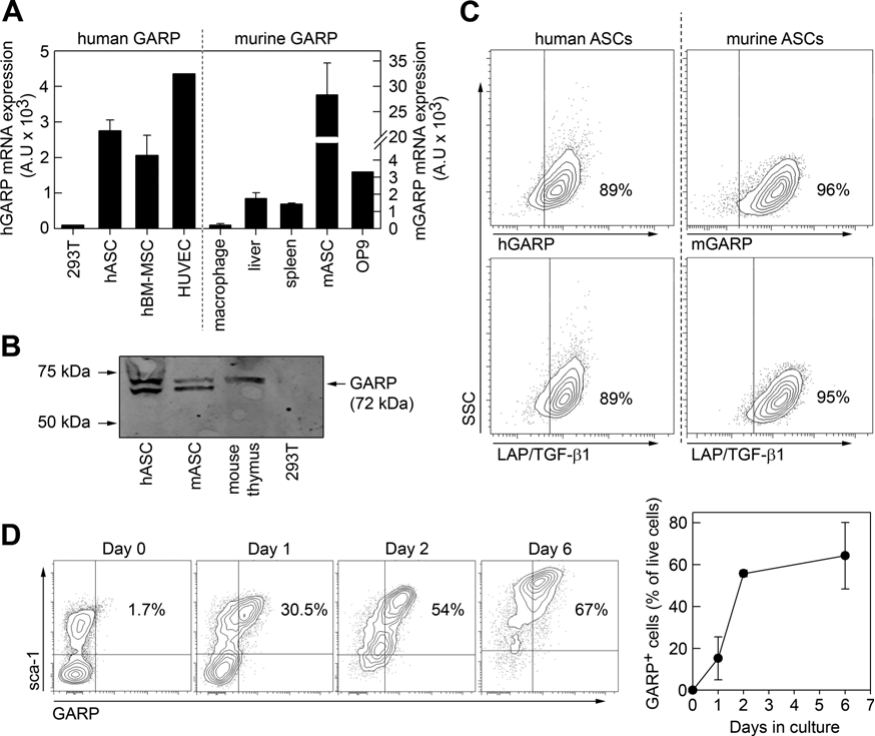
MSCs express GARP and LAP/TGF-β1 on their surface. (A): RT-qPCR analysis of GARP expression in human (left) and mouse (right) MSCs. hASCs and hBM-MSCs were analyzed together with human negative (293T) and positive (HUVEC) cell lines. Murine MSCs from adipose tissue (mASCs) and the MSC-line OP9 are shown in the right panel. As controls for murine samples we used BM-derived macrophages, mouse liver, and spleen. (B): Total protein from hASCs, mASCs, murine thymocytes, and 293T cells were analyzed by Western blot using an anti-mouse/human GARP antibody. (C): EDTA-harvested human and murine ASCs were stained for GARP (top) and LAP/TGF-β1 (bottom) surface expression and analyzed by flow cytometry. (D): Fat tissue from BALB/c mice was treated with collagenase type I and the resulting cell suspensions were stained for GARP and sca-1 expression before (day 0) and 1, 2, and 6 days after in vitro culturing and analyzed by flow cytometry. (A) and (D) show mean (SEM) of three independent experiments; (B) and (C) show a representative experiments out of >3. Abbreviations: GARP, glycoprotein A repetitions predominant; hASC, human mesenchymal stromal cells from adipose tissue; hBM, human bone marrow; LAP, latency-associated peptide; MSC, mesenchymal stromal cell; TGF-β1, transforming growth factor-β1.

A report by Barbet et al. 31 described the presence of GARP mRNA in hMSCs using TaqMan low density arrays (TLDA); however, no further analysis has been published on GARP and MSCs. Since TGF-β1 is a key regulator of multiple cellular functions (proliferation, differentiation, and immunomodulation) and GARP binds TGF-β1, we hypothesized that GARP could play an important role in MSCs biology. We therefore studied whether GARP expression was a general property of MSCs (mouse and human) and examined its surface localization. As previously described, *GARP* mRNA was expressed in human MSCs but we also detected GARP expression in mouse MSCs from different origins (Fig. 1A and Supporting Information Fig. S2). GARP protein was detected in both human and mouse adipose-tissue-derived MSCs (ASCs) by Western blot (Fig. 1B). In addition to the 72 kDa GARP band observed in thymocytes, we also detected an additional lower band in ASCs. This could be due to differences in glycosylation and/or protein processing 15,32. By fluorescence-activated cell sorting (FACS) we showed that both mASCs and hASCs expressed GARP and LAP/TGF-β1 on their surface (Fig. 1C). Interestingly, mASC and hASCs also contained high levels of intracellular GARP (Supporting Information Fig. S3). Harvesting mASCs using TrypLE dramatically decreased the mbTGF-β1 staining (Supporting Information Fig. S4) and we therefore used PBS with 2 mM EDTA when detaching MSCs for the analysis of mbTGF-β1 expression. In order to investigate the GARP expression on freshly isolated mASCs, we stained cell suspensions obtained from collagenase-digested fat tissue for GARP expression before and after adherence to plastic. We found that GARP was not expressed on freshly isolated sca-1^+^ cells (Fig. 1D, day 0) but was rapidly induced on sca-1^+^ adherent cells upon in vitro culture (Fig. 1D, day 1), reaching a 20–30-fold induction after 1–2 days, respectively, although later passages generally expressed higher levels of GARP (Fig. 1D, day 6 and Fig. 1C). The GARP expression on mASCs was not affected by TrypLE or the collagenase type 1 treatment (data not shown). These data suggest that GARP is not expressed on the surface of mASCs under physiological conditions and that it is induced upon in vitro culturing.

### GARP Binds LAP/TGF-β1 to the Cell Surface of mASCs

**Figure 2:**
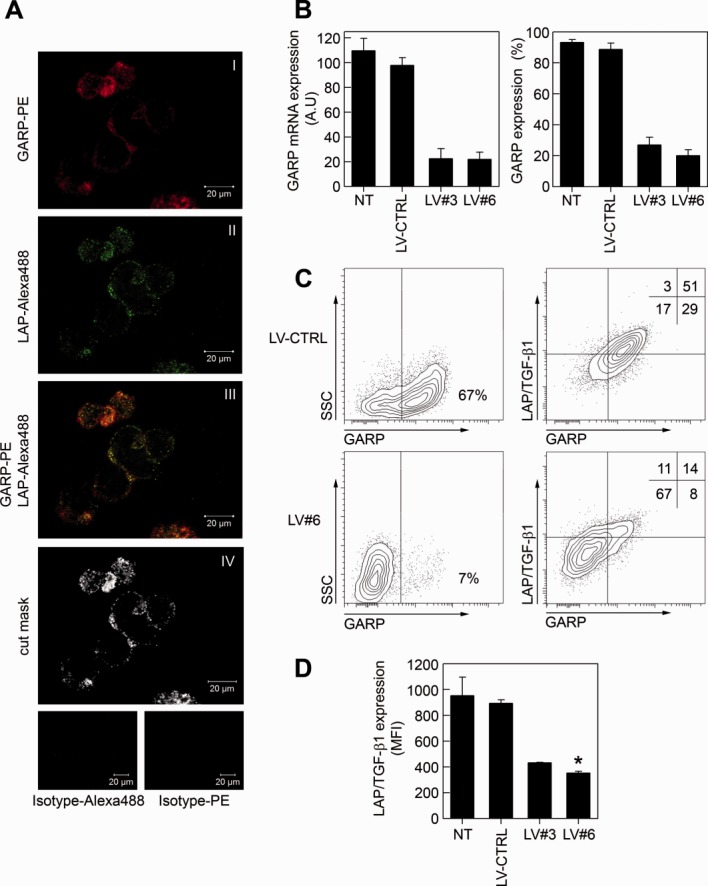
GARP binds LAP/TGF-β1 to the surface of mASCs. (A): Murine ASCs were surface stained for GARP-PE and LAP-Alexa488 or isotype controls (bottom panels) as described in Materials and Methods. Stainings are shown separately (panels I and II) and overlapping (panel III). A cut mask representing colocalized GARP/LAP/TGF-β1 is shown in panel IV. Cells were imaged using a Zeiss LSM 710 confocal microscope. (B): Murine ASCs were transduced with lentiviral vectors expressing a nonspecific shRNA (LV-CTRL) or GARP-specific shRNAs (LV#3 and LV#6) and GARP silencing in mASCs was assessed by qPCR (left panel) and flow cytometry (right panel). The general vector copy number/mASC for the different LVs used was two to three copies per cell. (C): LV-CTRL and LV#6 mASCs were stained for GARP (left panels) or GARP and LAP/TGF-β1 (right panels). One representative experiment out of three is shown. (D): The LAP/TGF-β1 expression levels (MFI) on NT, LV-CTRL, LV#3, and LV#6 mASCs were measured on a flow cytometer. Results are shown as mean (SEM) of three independent experiments. *, *p* < .05 versus LV-CTRL. Abbreviations: GARP, glycoprotein A repetitions predominant; LAP, latency-associated peptide; LV, lentiviral vector; TGF-β1, transforming growth factor-β1.

The main function described for GARP on T cells and platelets is the binding of LAP/TGF-β1 to their surfaces 17. We observed that GARP and LAP/TGF-β1 colocalized on mASCs using confocal microscopy (overlapping constant according to Mander = 0.65 ± 0.1), suggesting a close interaction (Fig. 2A). However, apart from GARP, other surface molecules, including neuropilin-1 and glucose-regulated protein 78 can bind LAP/TGF-β1 to the cell surface 33,34 and we found that both genes were expressed in mASCs (Supporting Information Fig. S5). In order to study the contribution of GARP to the surface expression of LAP/TGF-β1 on MSCs we knocked down GARP and analyzed the surface levels of LAP/TGF-β1 by flow cytometry. Mouse ASCs were transduced with LVs encoding for a nonspecific siRNA (LV-CTRL) or two different *GARP*-specific siRNAs (LV#3 and LV#6). Three to five days after transduction with LV#3 and LV#6, GARP expression, both at mRNA and protein level, was reduced by more than 80% compared to nontransduced (NT) and LV-CTRL mASCs (Fig. 2B). Importantly, GARP silencing significantly reduced the expression of LAP/TGF-β1 on the surface of mASCs (Fig. 2C, 2D) indicating that GARP participates in the attachment of mbTGF-β1 on the surface of mASCs.

### Silencing of GARP Increases the Secretion and Activation of TGF-β1

**Figure 3:**
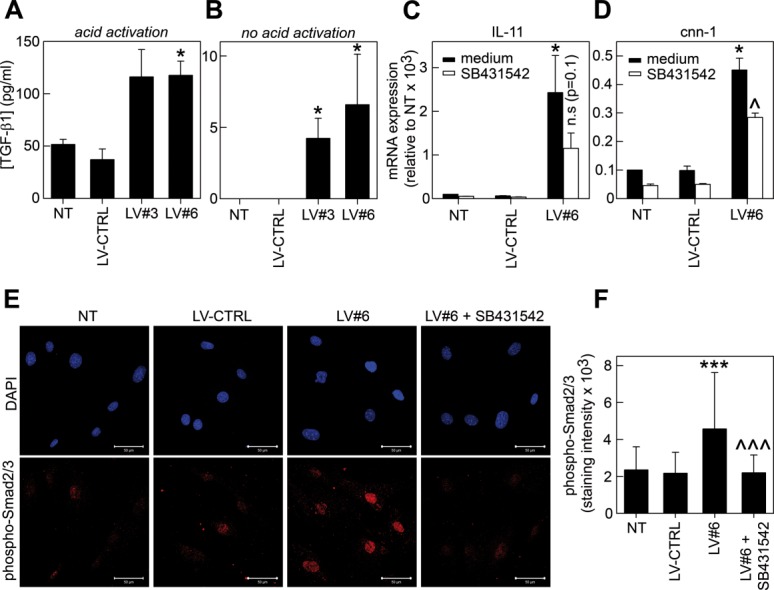
Glycoprotein A repetitions predominant modulates the secretion and activation of TGF-β1. (A, B): NT, LV-CTRL, LV#3, and LV#6 mASCs were cultured for 48 hours and the levels of TGF-β1 were measured in acid-activated (A) or non-acid treated (B) supernatants by ELISA. (C, D): NT, LV-CTRL, and LV#6 mASCs were cultured for 2 days in the presence (white bars) or absence (black bars) of SB431542 (10 µM). Total RNA was reverse transcribed and the expression levels of IL-11 (C) and cnn-1 (D) were analyzed by qPCR. Data are shown as mean (SEM) of four independent experiments. *, *p* < .05 versus LV-CTRL. ^, *p* < .05 versus LV#6 (medium). (E, F): NT, LV-CTRL, and LV#6 mASCs were cultured in eight-well chamber slides (5,000 cells/well) in medium supplemented with 0.2% FCS, with or without SB431542 (10 µM) for 48 hours. The cells were subsequently fixed, methanol permeabilized, and stained for phospho-Smad2/3 and mounted using Slowfade Gold antifade reagent (Life Technologies) containing DAPI. Images were captured using a Zeiss LSM 710 confocal microscope. One representative experiment out of two is shown. Data are represented as mean (SD). ***, *p* < .001 versus LV-CTRL; ^^^, *p* < .001 versus LV#6. Abbreviations: LV, lentiviral vector; TGF-β1, transforming growth factor-β1.

GARP has been shown to regulate the secretion of LAP/TGF-β1 23,35. We therefore analyzed the secretion of TGF-β1 by NT, LV-CTRL, LV#3, and LV#6 mASCs and found that GARP^−/low^ mASCs secreted significantly more TGF-β1 compared to NT and LV-CTRL mASCs (Fig. 3A). In contrast, the TGF-β1 mRNA levels were similar in all cell populations (Supporting Information Fig. S6B). GARP has also been implicated in the regulation of TGF-β1 activation by providing a cell surface platform for the presentation of LAP/TGF-β1 to integrins 23. We therefore measured the levels of active TGF-β1 in non-acid activated supernatants from NT, LV-CTRL, LV#3, and LV#6 mASCs. Low amounts of active TGF-β1 were found in supernatants from GARP^−/low^ mASCs but not in those from NT and LV-CTRL cultures (Fig. 3B). To corroborate the increased activation of TGF-β1 by GARP^−/low^ ASCs, we analyzed the expression of TGF-β1-inducible genes in NT, LV-CTRL, and GARP^−/low^ mASCs (transduced with LV#6). Addition of recombinant TGF-β1 induced the expression of interleukin (IL)−11 and to a lesser extent calponin (cnn)−1 in mASCs 36,37 (Supporting Information Fig. S6C). In agreement, we found that the expression of both genes was increased in GARP^−/low^ cells compared to NT and LV-CTRL cells (Fig. 3C, 3D). Addition of the TGF-β inhibitor SB431542 partially reduced the expression levels of both IL-11 and cnn-1 suggesting that autocrine TGF-β1 signaling is involved in their induction (Fig. 3C, 3D, white bars). If GARP^−/low^ mASC produce more active TGF-β1 we should observe an increase in Smad2/3 phosphorylation in these cells. Indeed we observed a clear increment in the levels of nuclear phospho-Smad2/3 in GARP^−/low^ mASCs that was blocked by the addition of SB431542 (Fig. 3E, 3F). Taken together, these results indicate that GARP regulates the secretion and activation of TGF-β1 by MSCs.

Finally, TGF-β1 is known to inhibit adipogenic differentiation of MSCs in vitro 9. However, while we found that GARP^−/low^ mASCs differentiated significantly less into adipocytes we could not increase adipogenesis by adding SB431542 suggesting that the block in adipogenesis does not depend on TGF-β1 (Supporting Information Fig. S7A, S7B).

### Silencing of GARP Reduces mASC Proliferation

**Figure 4:**
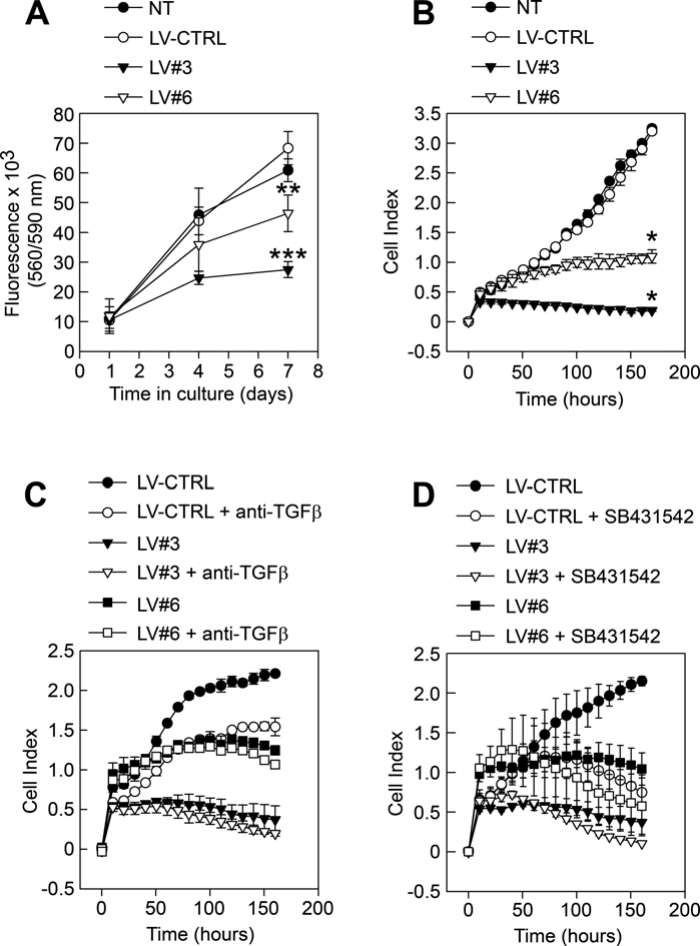
Silencing of glycoprotein A repetitions predominant affects the proliferation of mASCs. (A, B): NT (filled circles), LV-CTRL (open circles), LV#3 (filled triangles), and LV#6 (open triangles) mASCs were added (800 cells/well) to (A) 96-well plates and after 1, 4, and 7 days CellTiter-Blue were added to the cultures and fluorescence was measured at 560 nm or (B) to 16-well E-plates and proliferation was measured in real-time on an xCelligence real-time cell analyzer system. Results are presented as mean (SEM) of three independent experiments. *, *p* < .05 versus LV-CTRL; **, *p* < .01 versus LV-CTRL; ***, *p* < .001 versus LV-CTRL. (C, D): The proliferation of LV-CTRL, LV#3, and LV#6 mASCs with (open circles, triangles, squares, respectively) or without (filled circles, triangles, squares, respectively) anti-TGF-β1/2/3 Ab (C) and SB431542 (D) was measured in real-time on an xCelligence real-time cell analyzer system. Results are presented as mean (SEM) of two independent experiments. Abbreviations: LV, lentiviral vector; TGF-β1, transforming growth factor-β1.

GARP has been shown to influence the proliferation of T cells and cell lines with ectopic GARP expression 38,39. We thus set out to analyze the effect of the presence or absence of GARP on mASC proliferation in vitro. While the proliferation of ASCs transduced with LV-CTRL was similar to that of NT mASCs, transduction of mASCs with LV#3 and LV#6 decreased their proliferative capacity (Fig. 4A, 4B). Since TGF-β1 is known to modulate MSC proliferation we investigated whether the increased production of active TGF-β1 by GARP^−/low^ mASCs could be responsible for their decreased proliferation. However, the addition of a TGF-β1/2/3-neutralizing antibody or SB431542 to GARP^−/low^ mASCs did not promote but rather decreased the proliferation of mASC in general (Fig. 4C, 4D). These data suggest that the increase in TGF-β1 activation by GARP^−/low^ mASC is not responsible for their decreased proliferative capacity.

### Effect of mbTGF-β1 on the Immunomodulatory Capacities of ASCs

**Figure 5:**
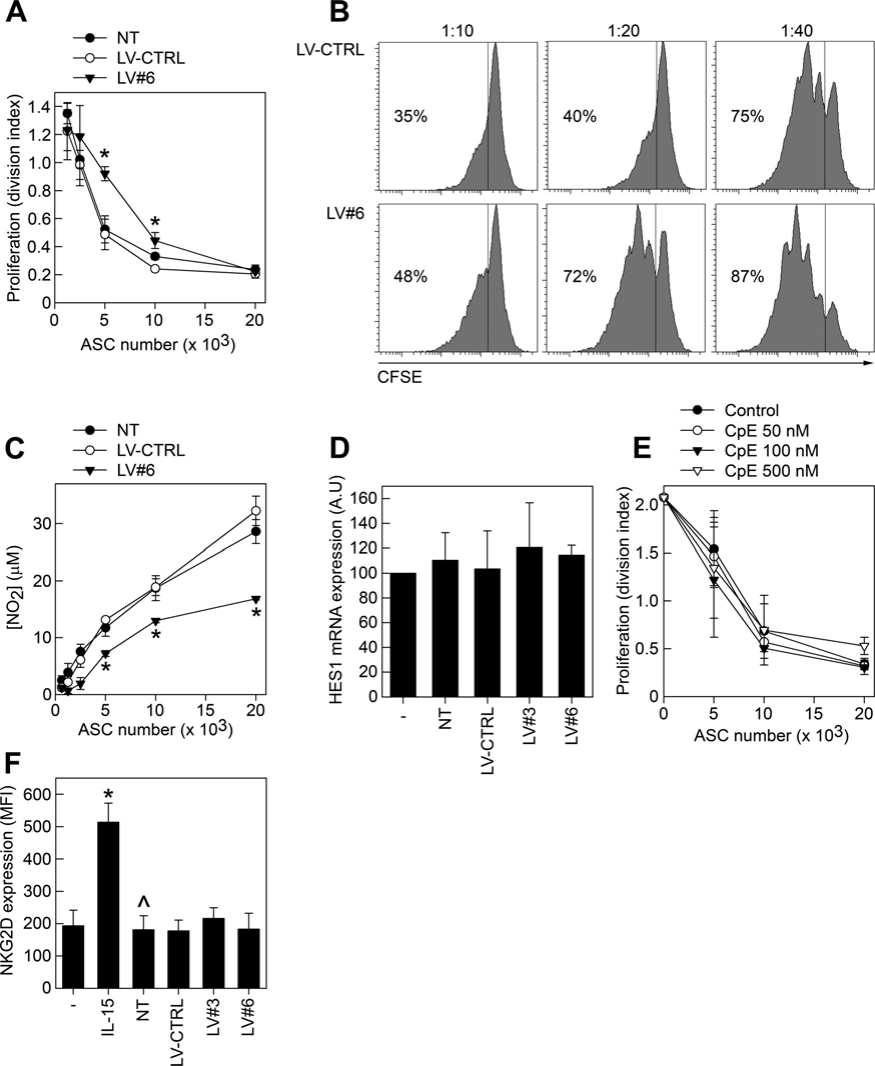
Role of glycoprotein A repetitions predominant in ASC-mediated immunomodulation. NT, LV-CTRL, and LV#6 mASCs were treated with mitomycin C and seeded at different numbers in 96-well plates. Balb/c splenocytes (200,000 cells/well) were labeled with CFSE and added to the ASC cultures and activated using anti-CD3 (1 µg/ml). After 72 hours, cells were harvested and the dilution of CFSE in CD4^+^ T cells were analyzed on a FACS Canto II cytometer using the FlowJo software. Results are plotted as (A) division index and (B) representative histograms of CFSE-dilution in CD4^+^ cells. Results are shown as mean (SEM) of three independent experiments. *, *p* < .05 versus LV-CTRL. (C): Supernatants from the MSC:splenocyte cocultures were analyzed for NO_2_ contents by Griess assay. Results are shown as mean (SEM) of three independent experiments. *, *p* < .05 versus LV-CTRL. As control, we set up parallel mASC cultures (LV#6, NT, and LV-CTRL) without splenocytes and detected similar cell numbers/viability (data not shown). (D): Splenocytes were cultured alone or with mitomycin C-treated NT, LV-CTRL, LV#3, and LV#6 mASCs for 24 hours in the presence of anti-CD3 (1 µg/ml). The splenocytes were subsequently assayed for their expression of HES1 by quantitative RT-PCR. (E): Mitomycin C-treated mASCs, at different numbers, were cocultured with CFSE-labeled splenocytes in the presence or absence of various concentrations of compound E (CpE). After 72 hours, cells were harvested and the dilution of CFSE in CD4^+^ T cells was analyzed on a FACS Canto II cytometer using the FlowJo software. (F): NT, LV-CTRL, LV#3 and LV#6 mASCs were treated with mitomycin C and seeded at 60,000 cells/well in a 24-well plate. Natural killer (NK) cells (60,000 cells/well) were added on top of the mASCs and activated using 100 ng/ml rmIL-15 for 5 days. CD49b^+^ NK cells were analyzed for their expression of NKG2D on a flow cytometer. Results are shown as mean (SEM) of three independent experiments. *, *p* < .05 versus NK cells alone, ^, *p* < .05 versus NK cells alone stimulated with IL-15. Abbreviation: LV, lentiviral vector.

Membrane bound TGF-β1 has been show to participate in the suppression of T-cell responses, induction of Tregs, and anergy in NK cells 17,19,22. Thus, we wanted to assess a possible role of mbTGF-β1/GARP in MSC-mediated immunomodulation. We found that silencing of GARP significantly reduced the capacity of mASCs to inhibit T-cell proliferation in vitro (Fig. 5A, 5B). We also measured the levels of NO_2_ in the supernatants of the suppression cultures and found that the NO_2_ levels were significantly lower in splenocyte:LV#6 cocultures compared to NT and LV-CTRL cocultures (Fig. 5C).

Ostroukhova et al. demonstrated that mbTGF-β1, but not soluble TGF-β1, induces Notch-HES1 signaling in responder T cells 40. However, HES1 expression was not induced in T cells upon coculture with mASCs and we could not detect any difference in HES1 expression in T cells cultured with GARP^−/low^ or with GARP^+^ mASCs. Furthermore, addition of compound E (CpE), an inhibitor of Notch signaling, to the cocultures did not affect the mASC-mediated suppression. Taken together, these data suggest that mASCs do not suppress T-cell responses via the Notch1 pathway (Fig. 5D, 5E).

MSCs can also inhibit the function of NK cells and we therefore analyzed whether mbTGF-β1 expression on mASCs could play a role in the control of NK cell activation. In contrast to the effect of GARP expression on MSC-mediated T-cell suppression, we found that GARP^−/low^ mASCs were equally efficient in inhibiting NK cell activation based on the upregulation of the NK cell activating receptor NKG2D in vitro (Fig. 5F).

### GARP Controls TGF-β1 Bioavailability and Proliferation of Human ASCs

**Figure 6:**
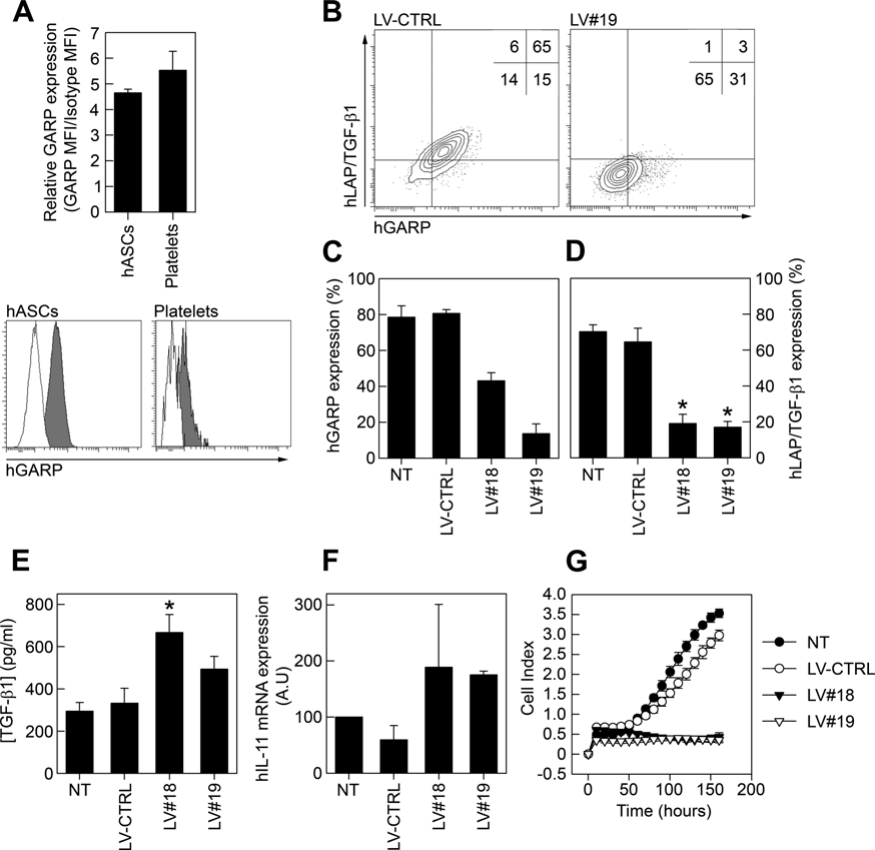
GARP expression on human ASCs. (A): Human ASCs and human platelets were stained for GARP expression and analyzed on a flow cytometer. In order to compare the relative GARP levels on hASCs and platelets we calculated the GARP MFI/Isotype MFI ratio (upper panel). GARP (gray histograms) and isotype control (white histograms) stainings of hASCs and platelets are shown (lower panel). (B–D): Human ASCs were transduced with lentiviral vectors expressing a nonspecific shRNA (LV-CTRL) or human GARP-specific shRNAs (LV#18 and LV#19). The general vector copy number/hASC for the LVs used was one to two copies per cell. (B): Representative dot plots of LAP/TGF-β1/GARP costaining on LV-CTRL (left) and LV#19 (right) hASCs are shown. (C, D): A quantification of GARP (C) and LAP/TGF-β1 (D) expression levels on NT, LV-CTRL, LV#18, and LV#19 hASCs are shown as mean (SEM) of three independent experiments. *, *p* < .05 versus LV-CTRL. (E, F): NT, LV-CTRL, LV#18, and LV#19 hASCs were cultured for 3 days and the levels of TGF-β1 in acidified supernatants were measured by ELISA (E) and the expression of IL-11 was analyzed by quantitative RT-PCR (F). Results are shown as mean (SEM) of two to four independent experiments. *, *p* < .05 versus LV-CTRL. (G): NT (filled circles), LV-CTRL (open circles), LV#18 (filled triangles), and LV#19 (open triangles) hASCs were added (800 cells/well) to 16-well E-plates and proliferation was measured on an xCelligence real-time cell analyzer system. Data are shown as mean (SD) of four experimental replicates. Abbreviations: GARP, glycoprotein A repetitions predominant; LAP, latency-associated peptide; LV, lentiviral vector; TGF-β1, transforming growth factor-β1.

We finally wanted to investigate whether GARP plays similar functions on hMSCs as compared to mMSCs. We first showed that hMSCs and human platelets contain comparable surface levels of GARP (Fig. 6A). As seen in mASCs, silencing of *hGARP* significantly decreased the expression of LAP/TGF-β1 on the surface of hASCs (Fig. 6B–6D). We also found that GARP^−/low^ hASCs secreted higher levels of TGF-β1 (Fig. 6E). Although we did detect an increase in IL-11 expression in GARP^−/low^ hASCs (Fig. 6F) we could not detect any increase in the levels of active TGF-β1 in non-acid-treated supernatants from these cells compared to NT and LV-CTRL hASCs (data not shown). Finally, as observed in mASCs, silencing GARP expression blocked proliferation of hASCs (Fig. 6G) and reduced the differentiation of hASCs into adipocytes (Supporting Information Fig. S7C). In summary, these data indicate that GARP plays a similar role for both murine and human ASCs.

## Discussion

A report by Barbet et al. 31 described the presence of *GARP* mRNA in hMSCs using TLDA which prompted us to study its potential role in MSC biology. In this manuscript, we have shown for the first time that murine and human MSCs express GARP on their surface. This finding could be relevant for the understanding of MSC biology since GARP is involved in the modulation of TGF-β1 localization/activity as well as in cell proliferation.

TGF-β1 is involved in a multitude of MSC functions, including proliferation, differentiation, and immunomodulation and it is thus important to understand the regulation of its production and activation by MSCs. TGF-β1 has classically been viewed as a secreted latent molecule which, through the association with LTBP, is targeted to the ECM where perturbations to ECM integrity (in response to injury and inflammation) result in the activation of TGF-β1 14,41. However, the discovery that GARP presents TGF-β1 on the surface of MSCs has opened up new possibilities. Many reports have shown that the cellular response to TGF-β differs depending on whether TGF-β is secreted or membrane bound 40,42,43. The reason for these differences is not well understood but mbTGF-β1 is thought to give rise to (a) high local concentration of active TGF-β1, (b) sustained TGF-β1 signaling, and (c) allowing other interactions to take place thereby modulating the response to TGF-β1 in a manner which secreted TGF-β1 cannot mimic 40,44. We found that GARP and LAP/TGF-β1 colocalized to a high degree on the surface of MSCs. Although other surface molecules also can bind LAP/TGF-β1, our *GARP* interference studies suggest that GARP is the main molecule involved in presenting mbTGF-β1 on MSCs.

Several reports indicate that GARP can modulate the secretion of LAP/TGF-β1 23,35. Our data clearly demonstrate that silencing GARP expression increased the secretion of LAP/TGF-β1 by MSCs. Interestingly, we found that GARP^−/low^ mASCs produced higher levels of active TGF-β1 compared to NT or LV-CTRL mASCs suggesting that GARP can modulate both the secretion and activation of TGF-β by MSCs. How the lack of GARP results in an increased activation of TGF-β by MSCs under normal culture conditions is not known. However, it has been shown that the LTBPs are important for TGF-β activation in vitro and in vivo 45–47 and GARP has been found to efficiently outcompete LTBP1 from binding to LAP/TGF-β1 23. Importantly, it has been shown that TGF-β1 activation by MSCs depends on LTBP-3 in vitro 48. We propose that in the absence of GARP, more LTBP/LAP/TGF-β1 complexes are secreted by the MSCs, resulting in an increased ECM-localization and subsequent steady state activation of TGF-β1 (Supporting Information Fig. S6A). Future studies should address under which circumstances and through which mechanisms mbTGF-β1 can be activated on MSCs.

Both murine and human GARP^−/low^ ASCs exhibited a significant reduction in their proliferative capacity. Since we observed an increased TGF-β1 activation by GARP^−/low^ ASCs and TGF-β1 is known to increase 49 or inhibit 50,51 the proliferation of MSCs we studied the possible involvement of TGF-β in reducing the proliferation of GARP^−/low^ ASCs. However, the addition of TGF-β neutralizing antibodies or SB431542 to GARP^−/low^ mASCs did not increase, but rather decreased their proliferation. This suggests that that TGF-β1 is a growth factor for MSCs and that the reduction in the proliferative capacity of GARP^−/low^ MSCs is TGF-β1 independent. How GARP can influence cell proliferation remains to be elucidated. In this direction, Zhou et al. demonstrated a positive correlation between GARP expression levels in HeLa cells and their proliferative capacity with an accumulation of the cell cycle-inhibitory proteins p53, p21/Cip1, and p27/Kip1 in GARP^low^ cells 39. In addition, some reports have detected the amplification of the chromosomal fragment 11q13, containing the GARP gene, in solid tumors again suggesting that GARP can control cell proliferation which is in agreement with our data 52,53. Another process that can be negatively affected by increased secretion of TGF-β1 is adipogenesis which was significantly reduced in GARP^−/low^ ASCs. However, the addition of SB431542 to the GARP^−/low^ ASCs during the differentiation process did not increase adipogenesis. The cell density of cultured MSCs can also affect their differentiation into adipocytes 54. Thus, our observed effect on MSC differentiation could be due to the lower proliferative capacity of GARP^−/low^ ASCs in combination with other unknown factors not related to TGF-β1.

Resting Tregs do not express GARP on their surface but they do contain high levels of intracellular GARP protein which upon activation relocalize to the surface membrane 22,32,38. Similarly, GARP was hardly expressed on freshly isolated cells from murine adipose tissue but its expression was rapidly induced on adherent cells with an MSC phenotype upon culturing in vitro. However, the circumstances that turn on GARP expression on MSCs are unknown and warrant further investigation. Interestingly, we found that MSCs expressed high levels of intracellular GARP (Supporting Information Fig. S3) and we are currently investigating the conditions that can lead to the activation/relocalization of GARP on MSCs. We would like to speculate that, as in Tregs, GARP surface localization could be an indicator of MSC activation.

As discussed before, the cellular response to TGF-β1 differs depending on whether TGF-β1 is secreted or membrane bound 40,42,43. Silencing of GARP on Tregs significantly decreased their suppressive activity in vitro while increasing the GARP expression enhanced their suppressive activity 21,38,55. MSCs have been shown to inhibit/modulate the activity of both innate and adaptive immune cells 56,57. Although GARP^−/low^ mASCs were equally efficient as NT mASCs in inhibiting NK cell activation, we found that GARP expression on mASC contributed to their ability to inhibit T-cell responses in vitro. The GARP^+^ mASCs were twice as effective as their GARP^−/low^ counterpart in inhibiting the proliferation of CD4^+^ T cells. This is an interesting finding considering the multitude of immunoregulatory molecules expressed by MSCs that can inhibit T-cell responses, including iNOS, PGE_2_, and growth factors 57. A possible mechanism of the increased suppression activity of GARP^+^ MSCs could involve mbTGF-β1-mediated induction of Notch1/HES1. However, our data showed that MSCs do not suppress T-cell responses via the Notch1 pathway. Interestingly, GARP^−/low^ mASCs produced less NO_2_ suggesting that the increased secretion of TGF-β1 by these cells could inhibit iNOS induction in an autocrine manner as previously described by Xu et al. 58. Thus, we believe that GARP could participate in the MSC-mediated inhibition of T-cell proliferation by controlling their TGF-β1 secretion/activation rather than directly interacting with T cells through mbTGF-β1 in a cell contact-dependent manner. However, the exact role of GARP remains to be clarified and whether MSCs, through their expression of GARP/mbTGF-β1, also can modulate T-cell differentiation into Treg and Th17 cells remains to be investigated. Also it is not known whether expression mbTGF-β1 could protect injected MSCs from NK cell-mediated lysis in vivo or contribute to the hypoimmunogenicity displayed by MSCs. Future studies on the role of GARP for the therapeutic efficacy of MSCs in vivo need to be performed in order to address the above mentioned issues.

In summary, we propose a model where quiescent GARP^−/low^ MSCs become activated upon injury acquiring surface GARP expression. GARP will arm the MSCs with mbTGF-β1, enhance their proliferative potential, and increase their ability to suppress T-cell responses. We propose GARP as a marker to identify activated/semiactivated MSCs and as a tool to enhance the therapeutic potency of MSCs.

## Conclusions

We have shown for the first time that mouse and human MSCs express GARP, a molecule previously found to be critical for anchoring latent TGF-β1 to the plasma membrane of activated Tregs and platelets. GARP appears to be the main protein presenting TGF-β1 on the surface of MSCs regulating its secretion and activation. GARP expression is required for MSC proliferation in a TGF-β1-independent manner. Finally, GARP enhances the immunomodulatory capacity of MSCs. We envision GARP as a new MSC marker of importance for the understanding of MSC biology and therapeutic activity.

## Notes

See http://www.StemCells.com for supporting information available online.
