# Supplementary figures and images for: Mesenchymal Stromal Cells Express GARP/LRRC32 on Their Surface: Effects on Their Biology and Immunomodulatory Capacity
Source: Stem Cells. 2014 Dec 18;33(1):183–95. doi: 10.1002/stem.1821 (PMC4309416; doi:10.1002/stem.1821)

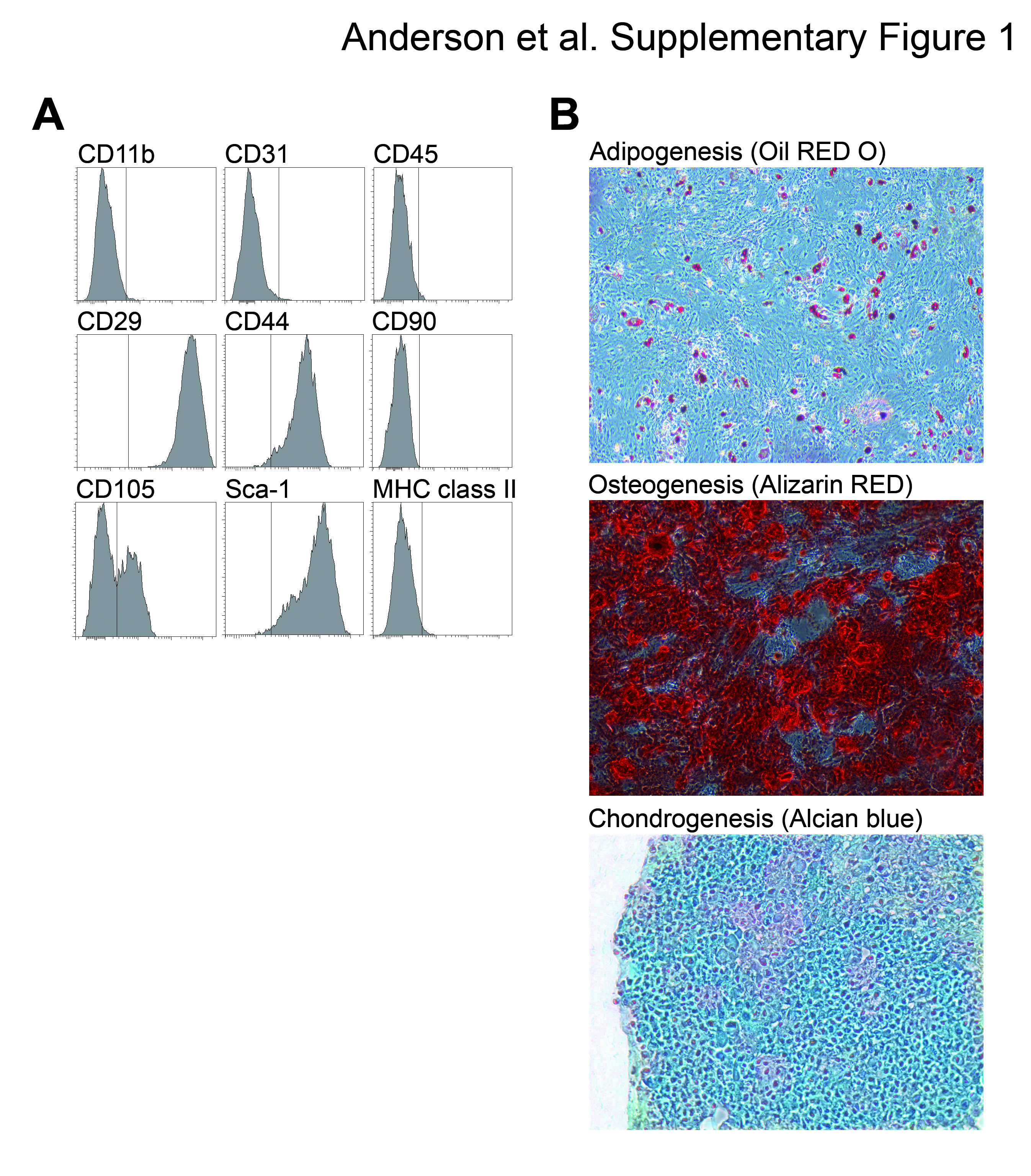

Supplement: Supplementary file 1 [file stem0033-0183-sd1.tif]

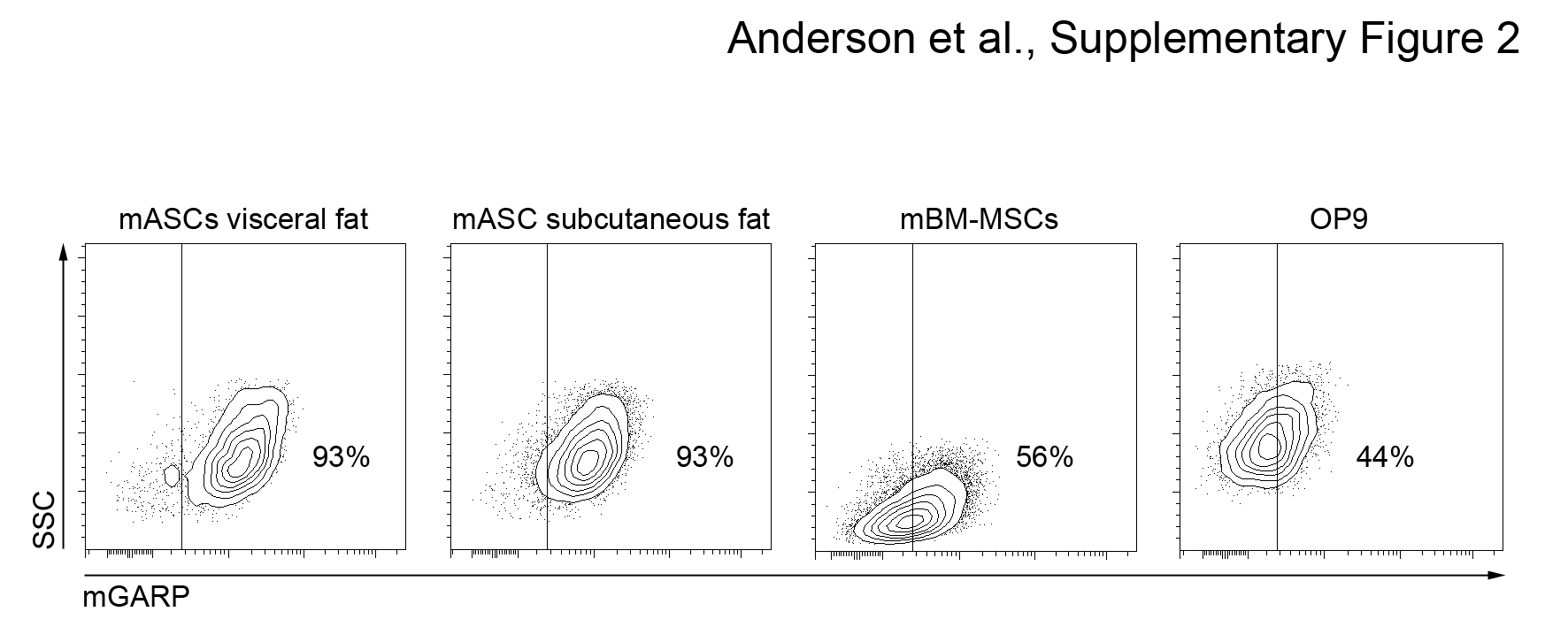

Supplement: Supplementary file 2 [file stem0033-0183-sd2.tif]

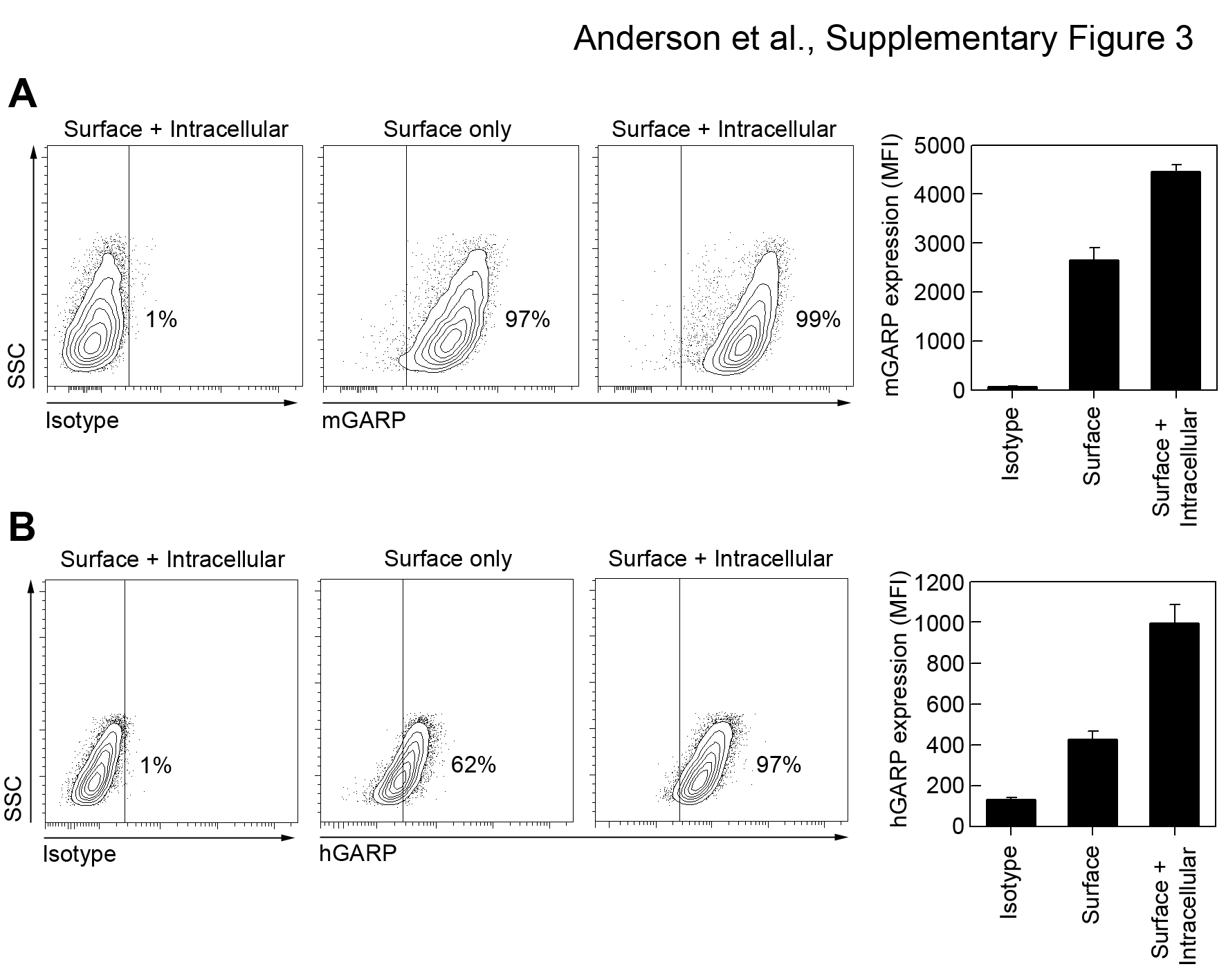

Supplement: Supplementary file 3 [file stem0033-0183-sd3.tif]

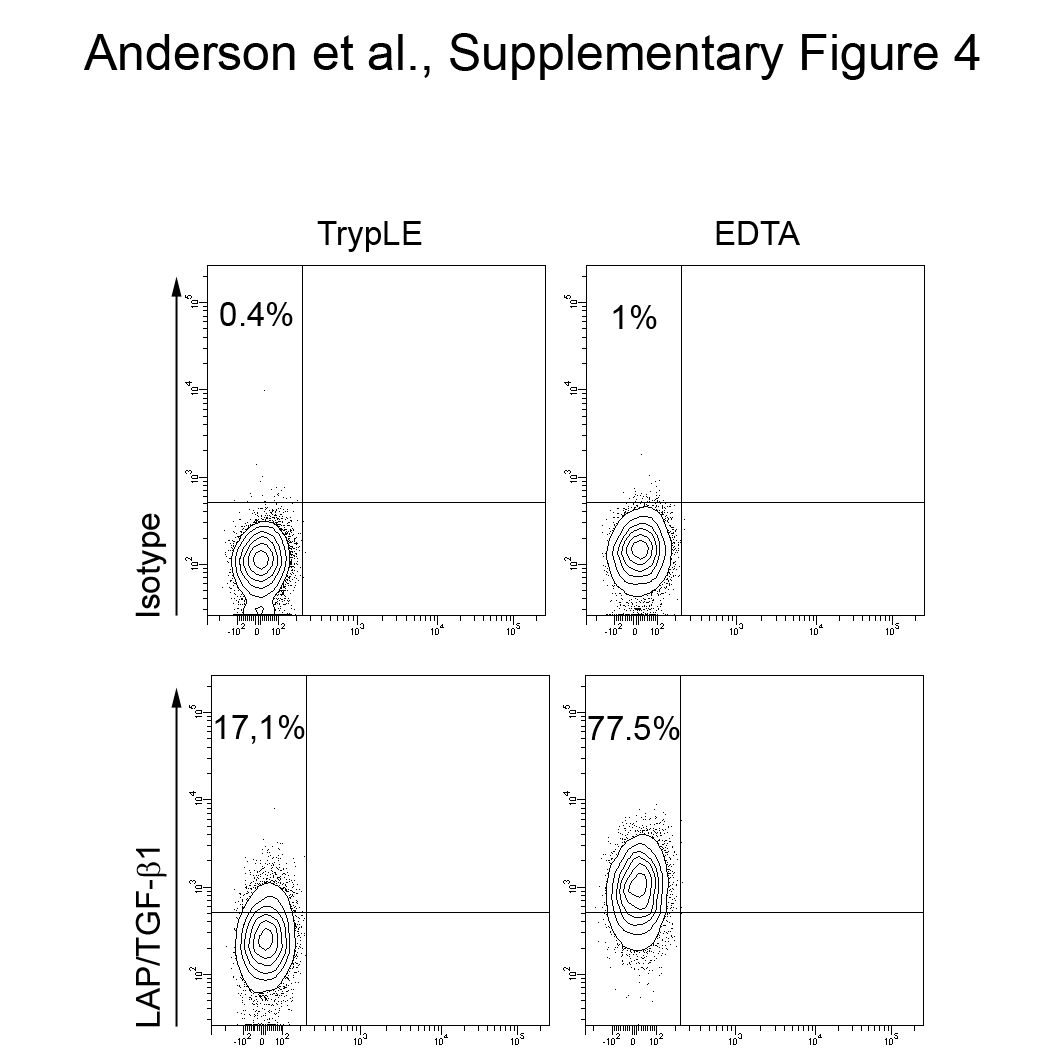

Supplement: Supplementary file 4 [file stem0033-0183-sd4.tif]

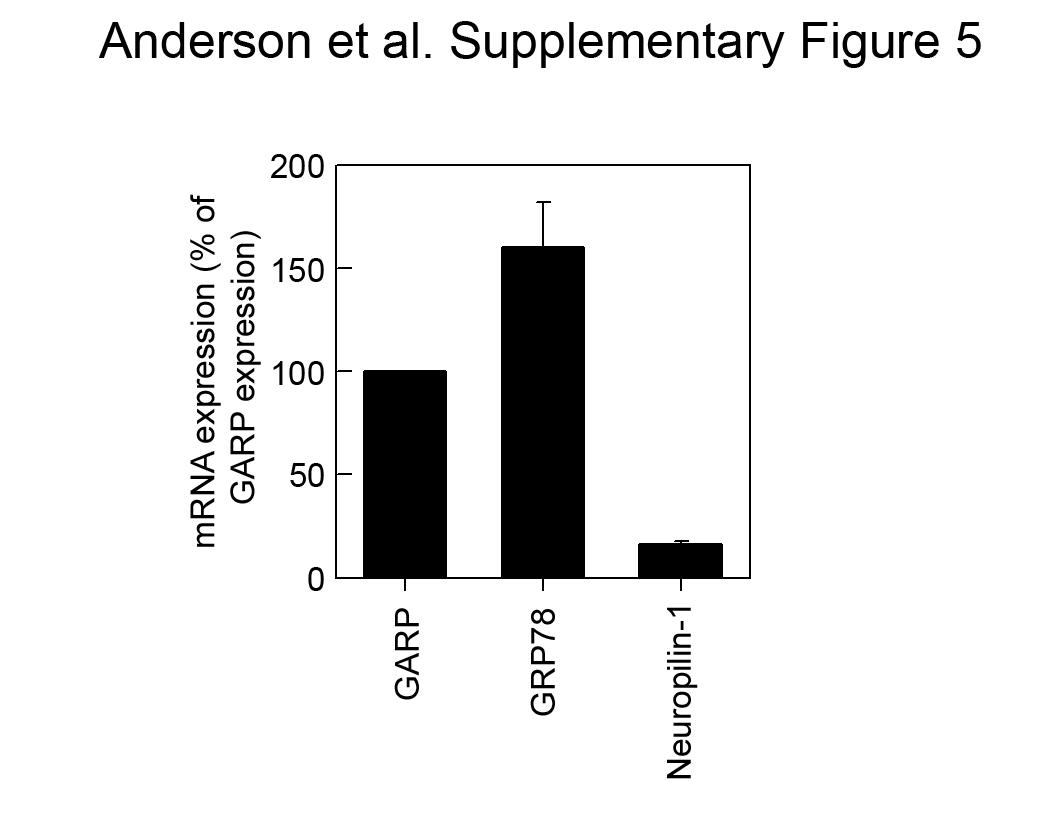

Supplement: Supplementary file 5 [file stem0033-0183-sd5.tif]

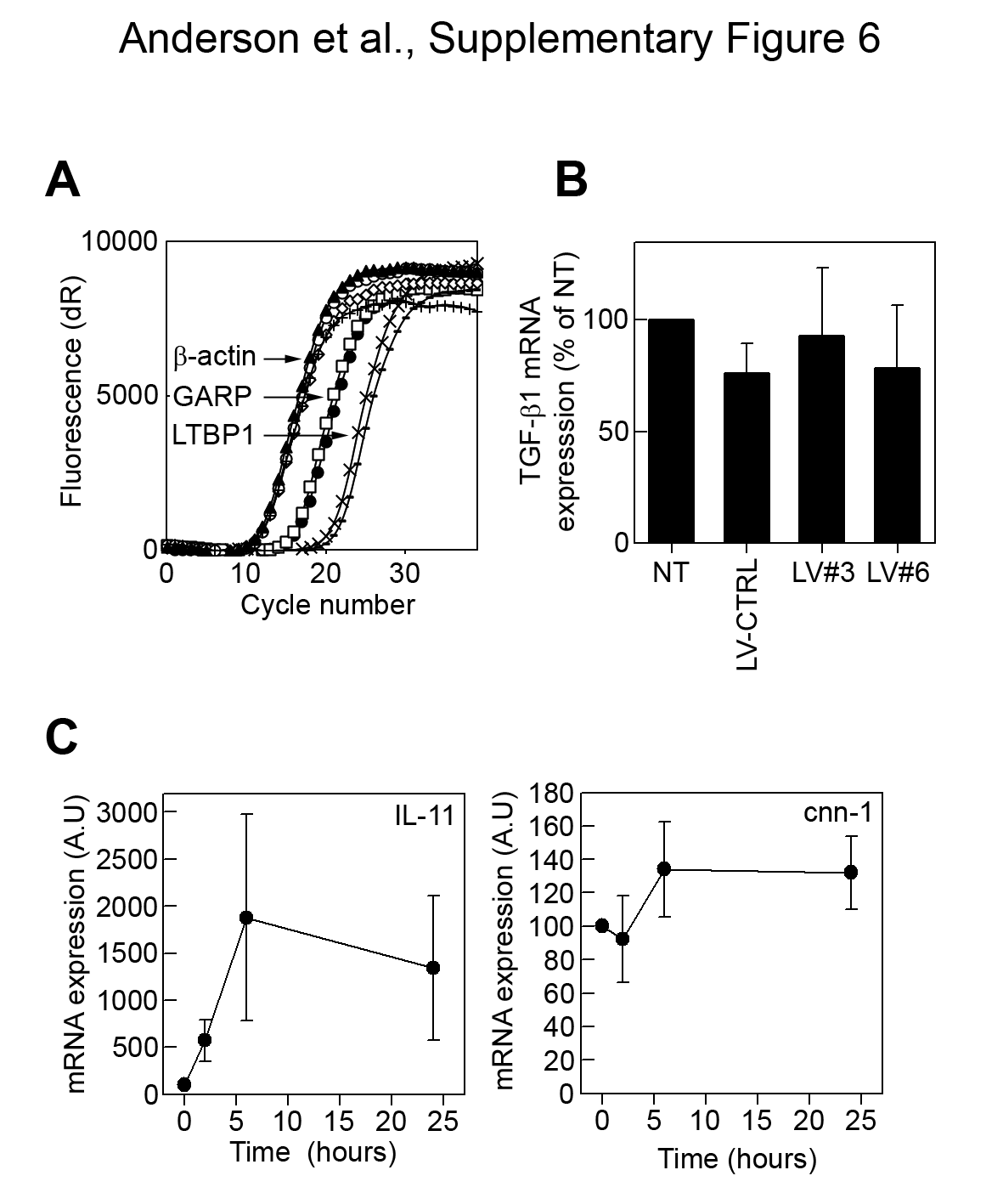

Supplement: Supplementary file 6 [file stem0033-0183-sd6.tif]

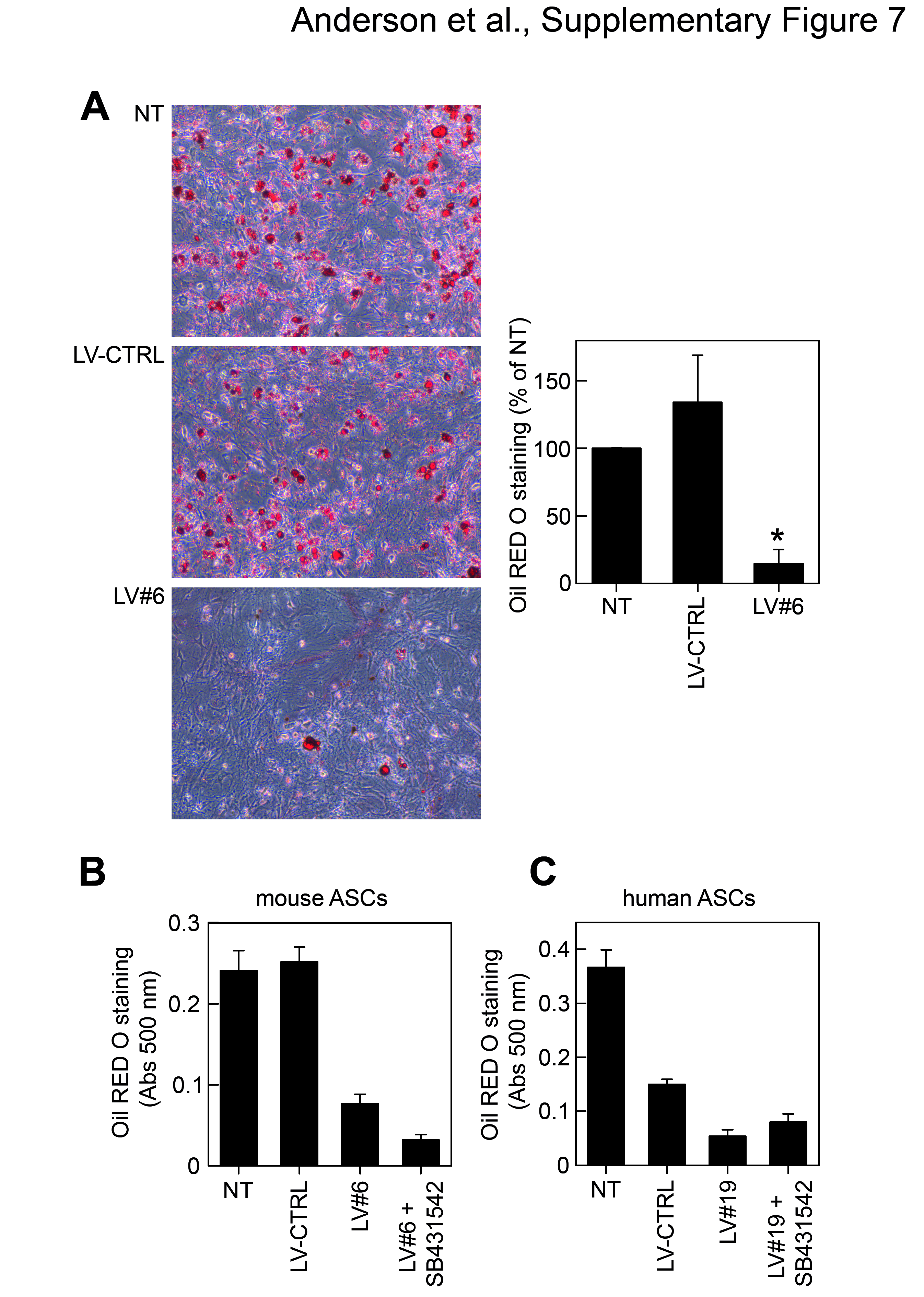

Supplement: Supplementary file 7 [file stem0033-0183-sd7.tif]
