# Supplementary material for: Mesenchymal Stromal Cells Express GARP/LRRC32 on Their Surface: Effects on Their Biology and Immunomodulatory Capacity
Source: Stem Cells. 2014 Dec 18;33(1):183–95. doi: 10.1002/stem.1821 (PMC4309416; doi:10.1002/stem.1821)
Supplement: Supplementary file 8 [file stem0033-0183-sd8.docx]

***Supplementary Figure 1. Phenotypic characterization and differentiation capacity of murine ASCs.***

(A) Murine ASCs were stained for a panel of markers used to identify MSCs and analyzed on a flow cytometer. (B) MSCs were induced to differentiate into adipocytes (Oil RED O staining), osteocytes (Alizarin Red staining) and chondrocytes (Alcian blue staining).

***Supplementary Figure 2. Expression of GARP on murine MSCs from different origin***

Murine ASCs isolated from visceral fat (epididymal fat pads), subcutaneous fat (inguinal fat pads), BM-MSCs and OP9 cells were stained for GARP and analyzed by flow cytometry.

***Supplementary Figure 3. Analysis of intracellular GARP expression in mouse and human ASCs***

Mouse (A) and human (B) ASCs, without permeabilization (middle panels) or fixed and permeabilized (right panels), were stained for GARP and analyzed on a flow cytometer as described in materials and methods. The bar diagrams show the MFI of surface or surface/intracellular GARP staining on ASCs and the data is represented as mean (SEM) of 2-3 independent experiments.

***Supplementary Figure 4. Sensitivity of LAP/TGF-β1 staining to TrypLE***

Murine ASCs were harvested with TrypLE (left panels) or 2 mM EDTA in PBS (right panels) and stained for isotype control (top panels) or LAP/TGF-β1 (bottom panels) and analyzed on a flow cytometer.

***Supplementary Figure 5. Analysis of the expression of TGF-β1 binding molecules in mASC by RT-qPCR***

Messenger RNA from mASC was reverse transcribed and the expression of GARP, GRP78 and Neuropilin-1 was analyzed by RT-qPCR as described in materials and methods. Expression levels of GRP78 and Neuropilin-1 are shown relative to GARP. Data are shown as mean (SEM) of 4 independent experiments. Neuropilin-1 FW: 5´-ACCTACATCATCTTTGCACC-3´; Neuropilin-1 RV: 5´-TAGAACGCCTGAAGAGGAGC-3´; GRP78 FW: 5´-AGCAGGACATCAAGTTCTTG-3´; GRP78 RV: 5´-AAATACGCCTCAGCAGTCTC-3´.

***Supplementary Figure 6. Analysis of the expression of LTBP1, TGF-β1 and TGF-β1-inducible genes in mASCs.***

(A) GARP and LTBP1 expression in mASCs were analyzed by RT-qPCR. (B) The expression levels of TGF-β1 mRNA by NT, LV-CTRL, LV#3 and LV#6 mASCs were analyzed by RT-qPCR five days after LV transduction. (C) Expression of IL-11 (left panels) and cnn-1 (right panels) in mASCs stimulated with 10 ng/ml TGF-β1 for 0, 2, 6 and 24 hours were analyzed by RT-qPCR. Data represent mean (SEM) from 3 independent experiments. LTBP1 FW: 5´- AACCAGGGTTACAGAGCATC-3´, LTBP1 RV: 5´- AGCCCTTATTGTCGTTCAGC -3´.

***Supplementary Figure 7. The block in adipogenesis in GARP^-/low^ ASCs is independent of TGF-β1.***

(A) Adipogenic differentiation was induced in NT, LV-CTRL and LV#6 as described in materials and methods and cells were stained with Oil RED O. Representative images are shown on the left. Oil RED O was extracted as described in supplementary materials and methods and quantified at 500 nm. The bar diagram (right) shows mean (SEM) from 3 independent experiments. *=p<0.05. Similar quantification of OIL RED O from adipogenic differentiation cultures using (B) mouse NT, LV-CTRL, LV#6 and LV#6 + SB431542 and (C) human NT, LV-CTRL, LV#19 and LV#19 + SB431542.
