# Supplementary material for: Mesenchymal Stromal Cells Express GARP/LRRC32 on Their Surface: Effects on Their Biology and Immunomodulatory Capacity
Source: Stem Cells. 2014 Dec 18;33(1):183–95. doi: 10.1002/stem.1821 (PMC4309416; doi:10.1002/stem.1821)
Supplement: Supplementary file 9 [file stem0033-0183-sd9.docx]

***Adipogenic differentiation (supplementary material and method).***

NT, LV-CTRL, LV#3 and LV#6 mASCs or NT, LV-CTRL, LV#18 and #19 hASC4 days after transduction, were seeded at 28,000 cells/cm^2^ and adipogenesis was induced the following day, with or without the addition of SB431542 (10 μM) as previously described. Cells were maintained in parallel in complete MesenCult as controls for spontaneous differentiation. Adipocytes were visualized on day 11-14 using Oil RED O (Sigma-Aldrich). Oil RED O was extracted with 100% isopropanol and absorbance was measured at 500 nm.
